# Identification of Na^+^/K^+^-ATPase inhibition-independent proarrhythmic ionic mechanisms of cardiac glycosides

**DOI:** 10.1038/s41598-017-02496-4

**Published:** 2017-05-26

**Authors:** Cai Hong Koh, Jianjun Wu, Ying Ying Chung, Zhenfeng Liu, Rong-Rong Zhang, Ketpin Chong, Vladimir Korzh, Sherwin Ting, Steve Oh, Winston Shim, Hai-Yan Tian, Heming Wei

**Affiliations:** 1 National Heart Research Institute Singapore, National Heart Centre Singapore, Singapore, 169609 Republic of Singapore; 2 Guangdong Province Key Laboratory of Pharmacodynamic Constituents of TCM and New Drugs Research, College of Pharmacy, Jinan University, Guangzhou, 510632 P.R. China; 3 Zebrafish Translational Unit, Institute of Molecular and Cell Biology, Singapore, 138673 Republic of Singapore; 4 International Institute of Molecular and Cell Biology in Warsaw, 4 Ks. Trojdena Street, 02-109 Warsaw, Poland; 5 Bioprocessing Technology Institute, A*STAR (Agency for Science, Technology and Research), Singapore, 138668 Singapore; 6 Cardiovascular & Metabolic Disorders Program, Duke-NUS Medical School Singapore, Singapore, 169857 Republic of Singapore

## Abstract

The current study explored the Na^+^/K^+^-ATPase (NKA) inhibition-independent proarrhythmic mechanisms of cardiac glycosides (CGs) which are well-known NKA inhibitors. With the cytosolic Ca^2+^ chelated by EGTA and BAPTA or extracellular Ca^2+^ replaced by Ba^2+^, effects of bufadienolides (bufalin (BF) and cinobufagin (CBG)) and cardenolides (ouabain (Oua) and pecilocerin A (PEA)) on the L-type calcium current (*I*_Ca,L_) were recorded in heterologous expression Cav1.2-CHO cells and human embryonic stem cell-derived cardiomyocytes (hESC-CMs). BF and CBG demonstrated a concentration-dependent (0.1 to 100 µM) *I*_Ca,L_ inhibition (maximal ≥50%) without and with the NKA activity blocked by 10 µM Oua. BF significantly shortened the action potential duration at 1.0 µM and shortened the extracellular field potential duration at 0.01~1.0 µM. On the other hand, BF and CBG at 100 µM demonstrated a strong inhibition (≥40%) of the rapidly activating component of the delayed rectifier K^+^ current (*I*_Kr_) in heterologous expression HEK293 cells and prolonged the APD of the heart of day-3 Zebrafish larva with disrupted rhythmic contractions. Moreover, hESC-CMs treated with BF (10 nM) for 24 hours showed moderate yet significant prolongation in APD90. In conclusion, our data indicate that CGs particularly bufadienolides possess cytosolic [Ca^2+^]_i_- and NKA inhibition- independent proarrhythmic potential through *I*_Ca,L_ and *I*_Kr_ inhibitions.

## Introduction

Cardiac glycosides (CGs) are selective inhibitors of sodium-potassium adenosine triphosphatase (Na^+^/K^+^-ATPase or Na^+^/K^+^-pump or NKA)^1^. Members of the cardiac glycoside family share a similar structural motif consisting of a basic perhydrophenanthrene nucleus and an unsaturated lactone ring at C-17 position. The lactone moiety defines two main classes of these compounds. Bufadienolides (like bufalin (BF) and cinobufagin (CBG)) and possess a six membered butenolide ring whereas cardenolides (ouabain (Oua) and pecilocerin A (PEA)) possess a five membered pentadienolide ring^1, 2^.

NKA functions to move three sodium ions out of the cells and two potassium ions in. In cardiac myocytes, NKA inhibition causes an accumulation of cytosolic Na^+^ which in turn activates the reverse-mode of Na^+^/Ca^2+^-exchanger. This leads to an increase in cytosolic [Ca^2+^]_i_ which perturbs the CGs through positive inotropic effect^3–5^. Hence, CGs, like digoxin, has been used for decades as a cardiotonic drug for heart failure^5^. Interestingly, CGs also demonstrates anti-proliferation and anti-tumour potentials^1, 6–10^.

However, CGs have long been known for possessing strong proarrhythmic effects that could cause syncope and virtually all forms of cardiac arrhythmias in human associated with impaired atrial-ventricular conduction and enhanced automaticity^1, 5^. In cardiac myocytes, CG-induced accumulation of cytosolic [Ca^2+^]_i_ is known to accelerate [Ca^2+^]_i_-dependent inactivation (CDI) of the L-type Calcium channels (LTCC) and lead to the suppression of the LTCC currents (*I*_Ca,L_)^11, 12^, which could be responsible for the shortening of cardiac action potential (AP) duration (APD) in cardiac myocytes of various animal species^11, 13–16^ and the shortening of the cardiac field potential (FP) duration of human induced pluripotent stem cell-derived cardiomyocytes^17^. Moreover, increased [Ca^2+^]_i._ and SR Ca^2+^ load by NKA inhibition could activate Calcium-Calmodulin Kinase II (CaMKII)-induce phosphorylation of the ryanodine receptor (RyR) on the sarcoplasmic reticulum (SR) of cardiac myocytes and enhanced SR Ca^2+^ leak and exacerbate spontaneous cardiac activity^18, 19^. However, questions remain as whether NKA inhibition and [Ca^2+^]_i_ accumulation by CGs is fully responsible for the pro-arrhythmic electrophysiological changes. The dissociation between inotropic and proarrhythmic actions of CGs indicates that the proarrhythmic effects of CGs may not result solely from NKA inhibition^18–21^. For example, Chan Su (toad venom) and its key ingredient BF have demonstrated more potent cardiotoxicity over Oua as additional proarrhythmic effects were achieved by Chan Su or BF after the Na^+^/K^+^-ATPase activity was fully blocked by Oua^20, 22^.

**Figure 1:**
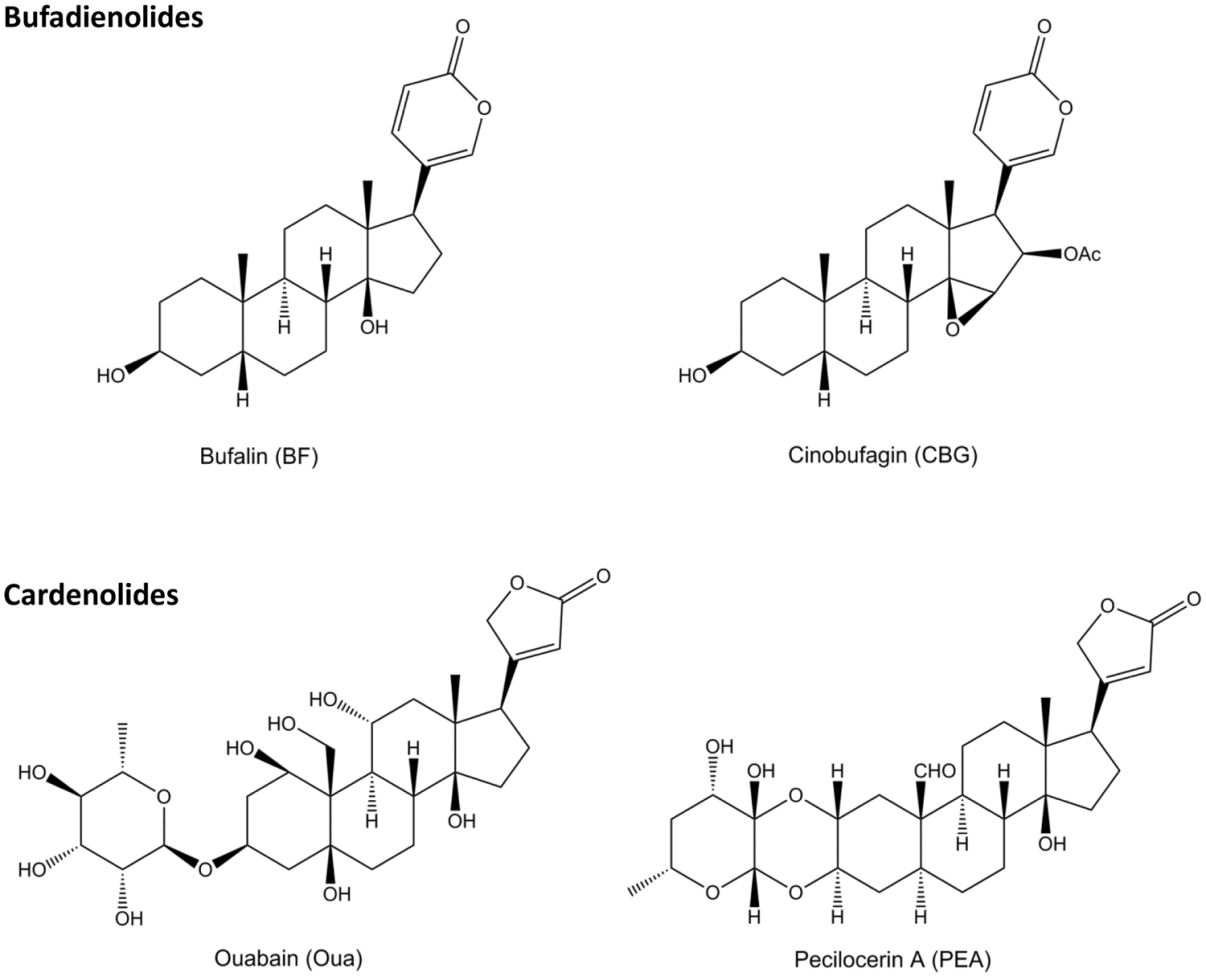
Structural formula of the 4 CGs adopted in the current study.

CG-induced cardiotoxicity remains to be a threat to human health and it may hamper their clinical potentials as hypertonic and anti-cancer drugs. Hence, it is important to fully understand the proarrhythmic effect and mechanisms of CGs. Here, we explored the potential [Ca^2+^]_i_- and NKA inhibition- independent proarrhythmic ionic mechanism of CGs. Bufadienolides (BF and CBG) and cardenolides (Oua and Pecilocerin A (PEA)) (Fig. 1 **)** with different NKA inhibition potencies^13, 21–24^, were assayed with the cytosolic Ca^2+^ chelated, extracellular Ca^2+^ or K^+^ depleted and NKA activity blocked. Effects of CGs on major cardiac ion channel currents and corresponding repolarization durations were assessed in heterologous expression systems, human embryonic stem cell (hESC)-derived cardiomyocytes (hESC-CMs) and zebrafish heart, respectively. Our data confirmed that, under physiological/toxicological concentrations, CGs particularly Bufadienolides like BF could either inhibit LTCC independently from intracellular [Ca^2+^]_i_ and NKA-inhibition, or, inhibited hERG at both high and low (prolonged effect) concentrations.

## Results

Based on that used in previous *in vitro* experiments^5–9^ and in patients^10^, the concentrations of BF, CBG, Oua and PEA adopted in the current study ranged from 0.1 to 100 µM, whereas 0.01~1.0 µM falls in the therapeutic range and 1.0~10 µM overlapped with the cardiotoxicity range.

### Cytosolic [Ca^2+^]_i_-independent effects of CGs on major cardiac ion currents

**Figure 2:**
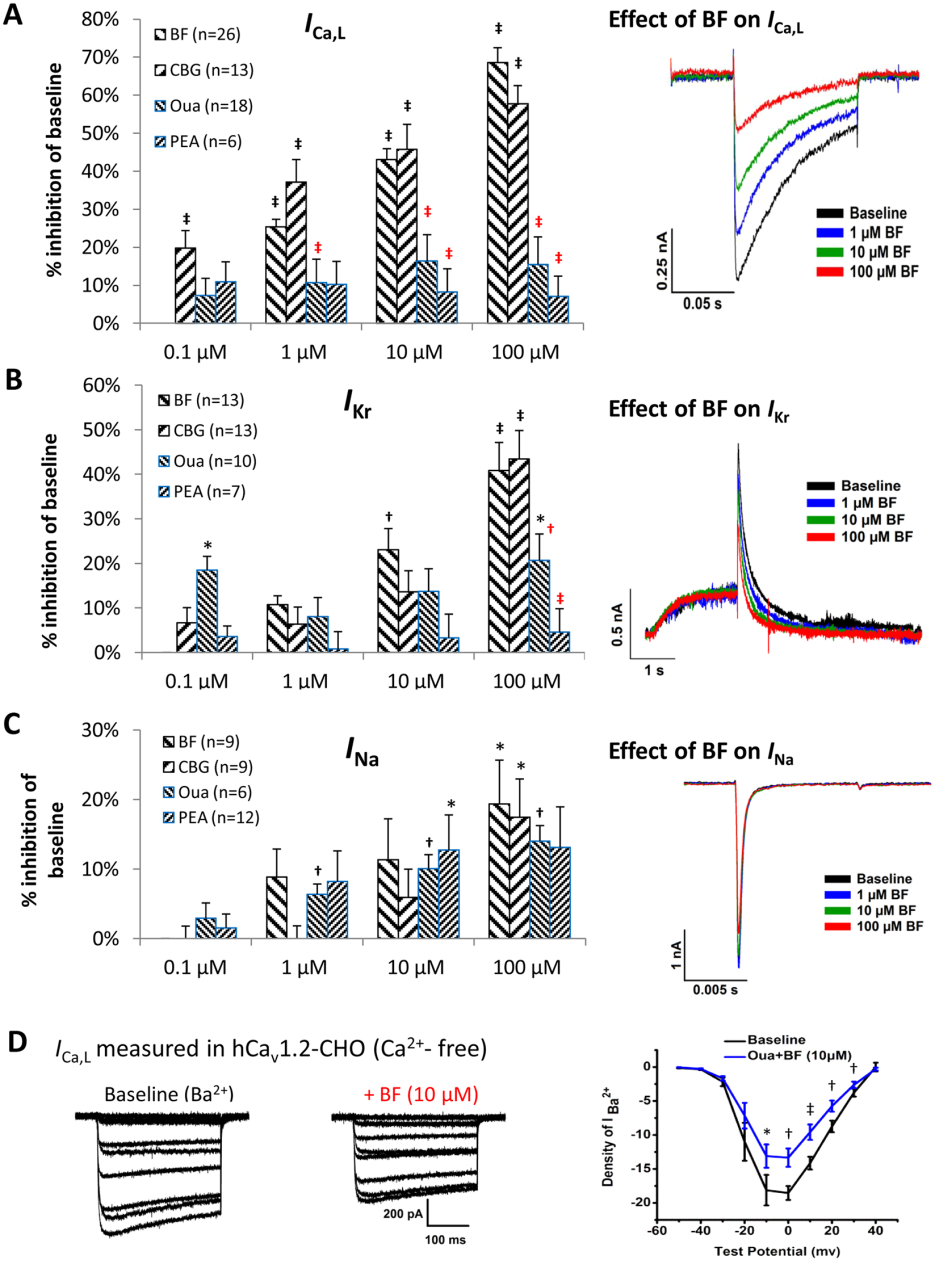
Effects of CGs on *I*_Ca,L_, *I*_Na_ and *I*_Kr_ in heterologous expression cell lines. (**A**) Effects of BF, CBG, Oua and PEA on *I*_Ca,L_ recorded from Cav1.2-CHO cells. (**B**) Effects of BF, CBG, Oua and PEA on *I*_Kr_ (peak tail current) recorded from hERG-HEK293 cells. (**C**) Effects of BF, CBG, Oua and PEA on *I*_Na_ recorded from Nav1.5-HEK293 cells. Shown on the left are the % inhibitions plotted in bar-graphs. Shown on the right are representative traces showing the effects of BF on *I*_Ca,L_, *I*_Na_ and *I*_Kr_ recorded by Patchliner®, the automated patch-clamping system. *p < 0.05, ^†^ *p* < 0.01, ^‡^ *p* < 0.001, vs. baseline of each CG (One-Way ANOVA). ^†(red)^ *p* < 0.01, ^‡(red)^ *p* < 0.001, vs. BF/CBG at same concentrations (Two-Way repeated measures ANOVA). Data are presented as mean ± SEM. (**D**) Effects of BF on *I*_Ca,L_ recorded in Cav1.2-CHO cells with Ca^2+^ depleted in the extracellular solution (recorded by conventional voltage-clamping). (Left) Representative traces of *I*_Ca,L_ recorded in a Cav1.2-CHO cell at baseline (Ca^2+^-free extracellular conditions achieved by using BaCl_2_ to replace CaCl_2_) and post 10 µM BF treatments. **(**Right) I-V curves showing the voltage-dependent *I*_Ca,L_ current densities (pA/pF). *p < 0.05, ^†^ *p* < 0.01, ^‡^ *p* < 0.001, vs. baseline.

Automated patch clamping was adopted to screen the impacts of CGs on major cardiac ion currents in heterologous expression cell lines. With the intracellular solutions contained 20 mM of EGTA and 5 mM BAPTA (to chelate the intracellular free Ca^2+^, BF and CBG demonstrated concentration-dependent (1~100 µM) inhibition of *I*_Ca,L_ over baseline (with maximized inhibition of ~70% and ~60%, and IC_50s_ of 12.5 µM and 15.3 µM, respectively) which were more remarkable than that of Oua and PEA (with maximized inhibition ~15% and ~10%, and estimated IC_50s_ of way above 100 µM). The concentration-dependent effects of BF and CBG on *I*_Ca,L_ inhibition were confirmed by One-Way ANOVA test showing significant intra-group (among different concentrations) differences (*p* < 0.001). In addition, BF and CBG produced a marked (>40%) inhibition of the rapidly activating component of the delayed rectifier K^+^ current (*I*_kr_) at 100 µM with estimated IC_50s_ at slightly above 100 µM compared to that of Oua and PEA which were way above 100 µM. On the other hand, all CGs demonstrated limited inhibitions (all <20%) on *I*_Na_ **(**Fig. 2 **)** with the estimated IC_50s_ way above 100 µM. Outcomes from Two-Way repeated ANOVA testing indicated that the effects of BF and CBG on *I*_Ca,L_, *I*_Kr_ and *I*_Na_, respectively did not differ from each other whereas the effects of BF and CBG on *I*_Ca,L_ at 1.0 µM, 10 µM and 100 µM were significantly stronger than Oua and PEA at the corresponding concentrations (*p* < 0.001). At 100 µM the inhibitory effects of BF and CBG on *I*_kr_ were much stronger than Oua and PEA (*p* < 0.001).

To evaluate the accuracy of our automated patch-clamping recording, the effects of BF and CBG on *I*_Kr_ were independently verified by Shanghai Institute of Materia Medica, Chinese Academy of Science, where hERG-CHO cells (different from hERG-HEK293 we used) and a Sophion QPatch Automated Patch Clamp Systems (Biolin Scientific, Stockholm, Sweden) were used. At 40 µM (the highest concentration used), BF and CBG suppressed *I*_kr_ by ~20% and ~40%, respectively, while Cisapride (a positive control) achieved a full blocking of hERG. Therefore, these results confirmed our findings on *I*_Kr_.

The cytosolic Ca^2+^-independent effects of BF on *I*_Ca,L_ were further validated in Cav1.2-CHO cells under Ca^2+^-free extracellular conditions (Fig. 2D). With the extracellular Ca^2+^ replaced by Ba^2+^ which carried the current through Ca^2+^ channels, application of 10 μM BF rapidly reduced *I*_Ca,L_ density by ~30%, suggesting that BF may possess a direct inhibitory effect on LTCC.

### NKA inhibition-independent effect of BF and CBG on *I*_Ca,L_

**Figure 3:**
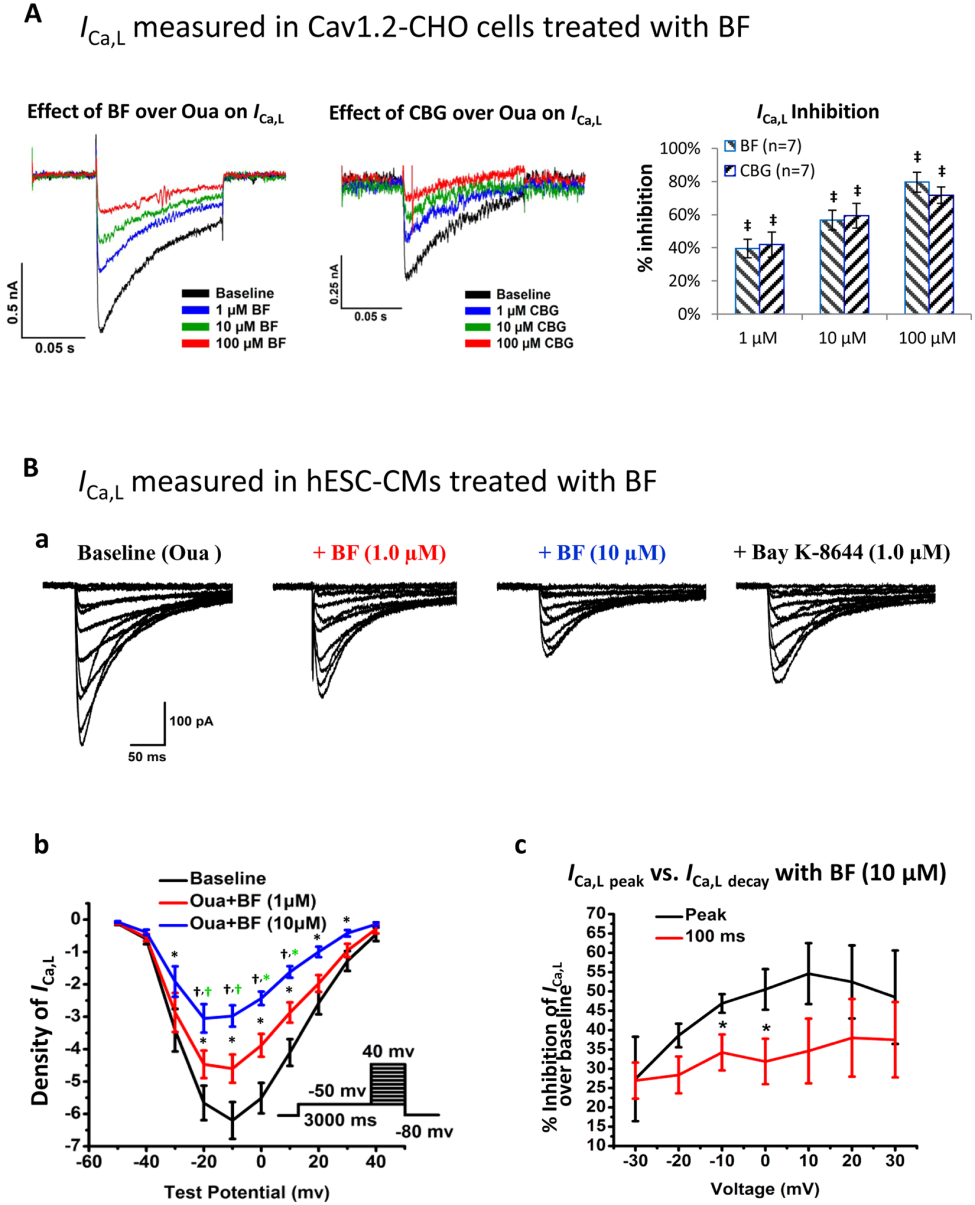
NKA inhibition-independent effects of BF on *I*_Ca,L_ in Cav1.2-CHO and hESC-CMs. (**A**) Effects of BF and CBG on *I*_Ca,L_ recorded in Cav1.2-CHO cells pre-incubated with 10 µM of Oua and with extracellular KCl replaced by CsCl to further suppress the NKA activity. Shown on the left and middle respectively are representative traces showing the effects of BF and CBG on *I*_Ca,L_ recorded by Patchliner®. Shown on the right is the % inhibition plotted in bar-graph. ^‡^ *p* < 0.001, vs. baseline (One-Way ANOVA). Data are presented as mean ± SEM. (**B**) Effect of BF on *I*_Ca,L_ in hESC-CMs with NKA fully blocked by Oua (recorded by conventional voltage-clamping). (a) Representative traces of the voltage-gated *I*_Ca,L_ recorded from a hESC-CM at baseline (with 10 µM Oua incubation for 10 minutes), with subsequently treated with 1.0 and 10 µM of BF for 5 minutes, and finally with Bay K8644 (1 µM) treatment. (b) I-V curves showing the voltage-dependent *I*_Ca,L_ current densities (pA/pF). *p < 0.05, ^†^ *p* < 0.01, vs. baseline (One-Way ANOVA); ^*(green)^ *p* < 0.05, ^†(green)^ *p* < 0.01, vs. 1.0 µM BF (One-Way ANOVA). n = 7. (c) Comparison of the *I*_Ca,L_ current densities at peak (*I*_Ca,L peak_) and during decay (*I*_Ca,L decay_) recorded from cells treated with BF (10 µM). Data presented are % inhibition over baseline levels of *I*_Ca,L_ at peak and at 100 ms during current decay. n = 16.

As it is well-received that NKA-inhibition by CGs is the cause of the increase cytosolic [Ca^2+^]_i_ and CDI^1, 3–5^, we further validated the role of NKA inhibition in the inhibitory effect of BF and CBG on *I*_Ca,L_. As Oua has been commonly adopted as a standard NKA inhibitor, we treated Cav1.2-CHO and hESC-CMs with 10 µM Oua which is known to completely block the NKA activity^20, 22^. As shown in Fig. 3A, BF and CBG concentration-dependently inhibited *I*_Ca,L_ in Cav1.2-CHO (with extracellular KCl replaced by CsCl to further suppress the NKA activity) with similar magnitudes as shown in Fig. 2A. The concentration-dependent effects of BF and CBG on *I*_Ca,L_ inhibition were confirmed by One-Way ANOVA test showing significant intra-group (among different concentrations) differences (*p* < 0.001). Moreover, BF (1.0 and 10 µM, CBG not tested) significantly inhibited *I*_Ca,L_ in hESC-CMs and the effect was reversed by Bay K8644 (1 µM), an opener of LTCC (Fig. 3Ba, Bb). Noticed that the magnitude of peak *I*_Ca,L_ inhibition was greater than that during the current decay (Fig. 3Bc).

### NKA inhibition-independent effect of BF on [Ca^2+^]_i_ transients in hESC-CMs

**Figure 4:**
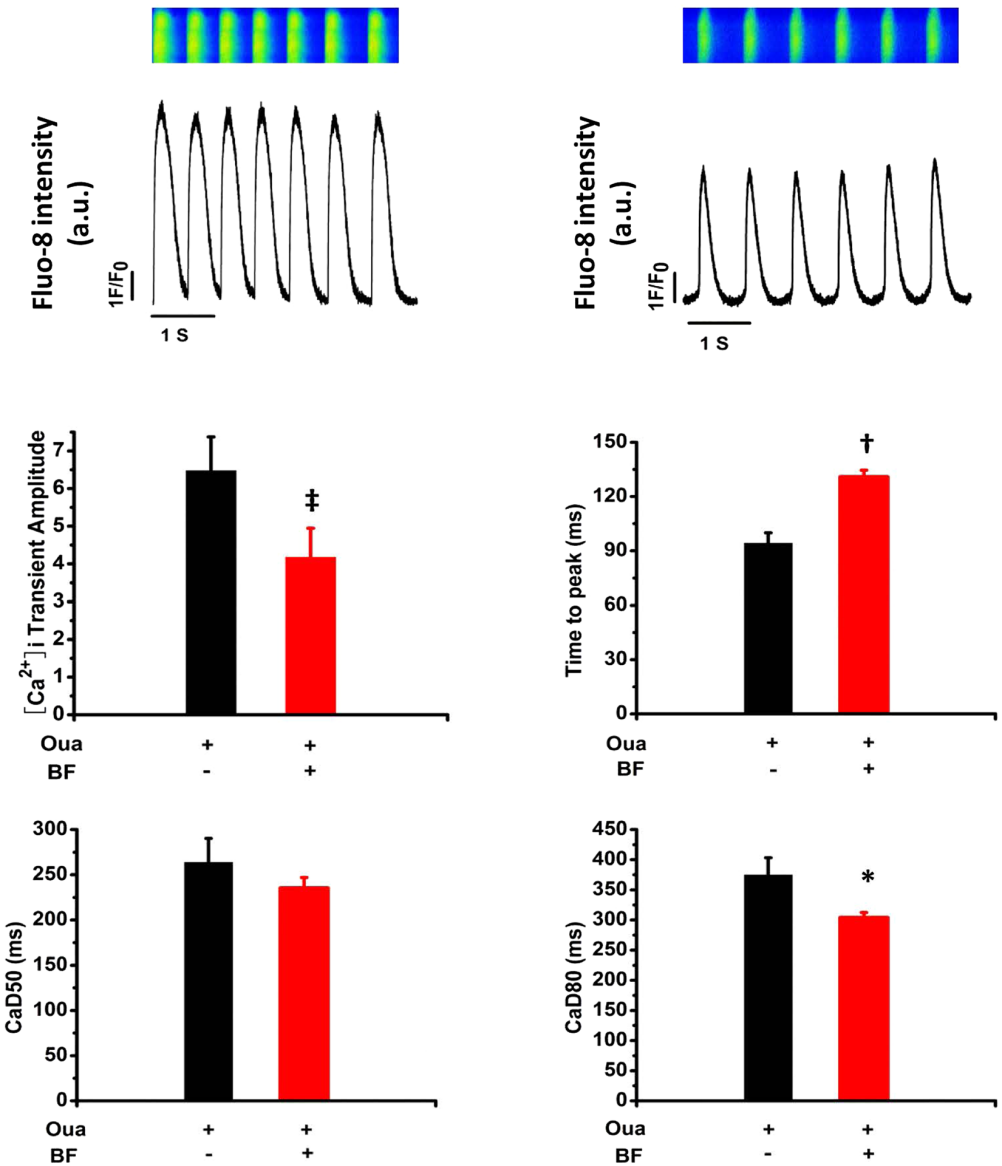
Effect of BF on [Ca^2+^]_i_ transients in hESC-CMs. Spontaneous [Ca^2+^]_i_ transients were recorded in Fluo-8 loaded hESC-CMs pre-treated with 10 µM Oua for 5 minutes followed by 10 µM BF. (**A**) Representative traces of [Ca^2+^]_i_ transients recorded in a cell at baseline (with Oua) and with subsequent exposure to BF. The relative [Ca^2+^]_i_ transients intensity is plotted as fluorescence ratio (F_1_/F_0_). (**B**) Bar-graphs show [Ca^2+^]_i_ transient amplitude, time to peak and duration (CaD) at 50% and 80% recovery (CaD50 and CaD80, respectively). ^*^ *p* < 0.05, ^†^ *p* < 0.01, ^‡^ *p* < 0.001, vs. baseline. n = 7.

[Ca^2+^]_i_ transients in hESC-CMs is facilitated by Ca^2+^-induced Ca^2+^ release (CICR) from SR which depends on the Ca^2+^ influx through LTCC. A previously report that Chan Su and BF were capable of suppressing [Ca^2+^]_i_ in neonatal rat cardiac myocytes with NKA activity fully inhibited by 10 µM Oua^20^. Similarly, we observed that on hESC-CMs pre-incubated with 10 µM Oua for 3 minutes, BF (10 µM) reduced the [Ca^2+^]_i_ transients amplitude (from 6.47 ± 0.90 to 4.18 ± 0.77, *p* < 0.001, n = 7) and duration at 50% and 80% recovery (CaD50 and CaD80), and increased time to peak (Fig. 4). Such findings support the inhibitory effect of BF on LTCC.

### Cytosolic [Ca^2+^]_i_-independent effects of CGs on APs of hESC-CMs

**Figure 5:**
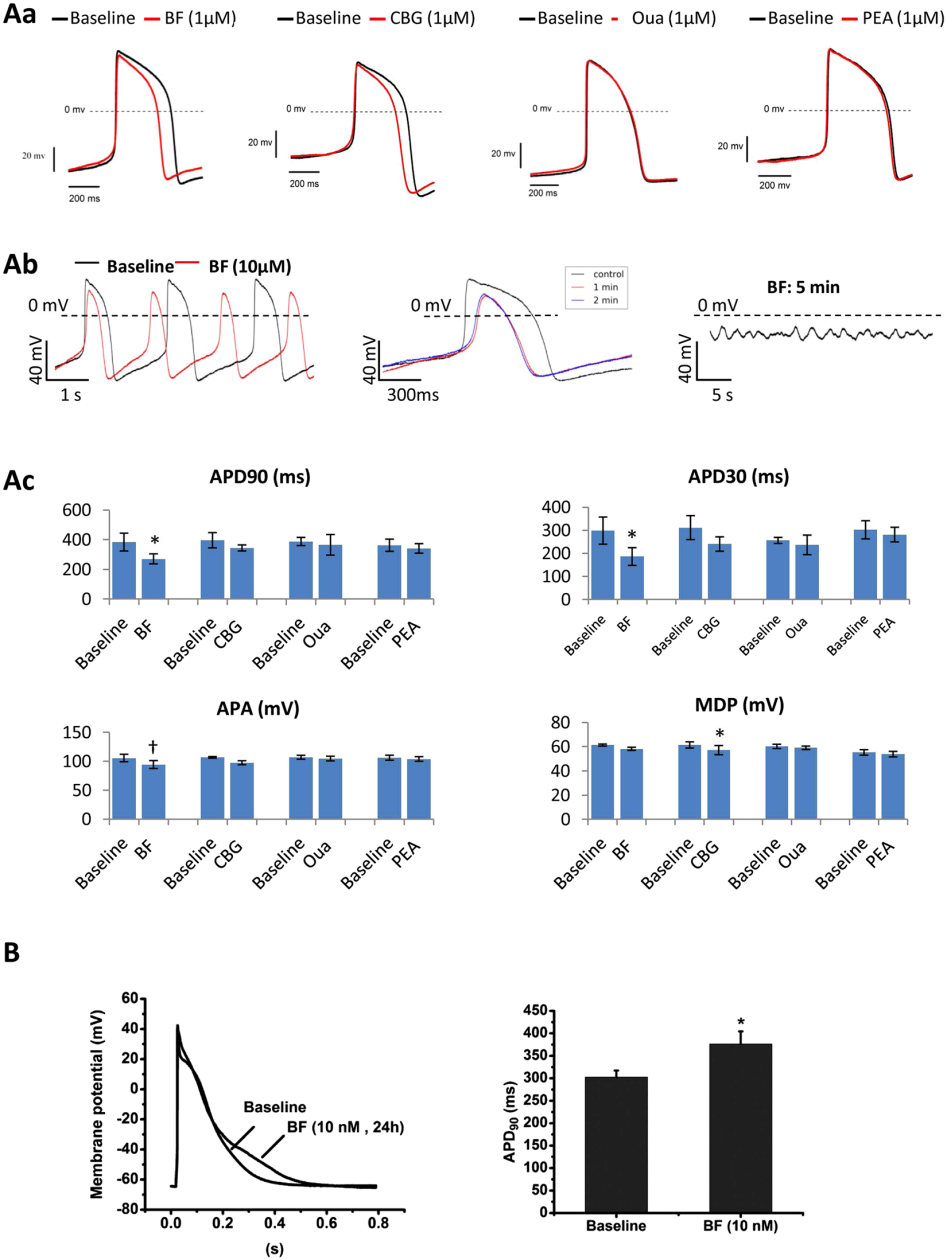
Effect of CGs on APs in hESC-CMs. Effects of CGs on APs were recorded in single hESC-CMs. (**A**a) Representative AP waveforms recorded in hESC-CMs exposed to 1 µM of BF, CBG, Oua and PEA for 1–2 minutes. (**A**b) Representative AP waveforms recorded in hESC-CMs treated with BF (10 µM) for different time points. (**A**c) Bar-graphs show APD90, APD30, APA and MDP measured in hESC-CMs 2 minutes post application of BF, CBG, Oua and PEA. ^*^ *p* < 0.05, ^†^ *p* < 0.01, vs. baseline (two-tailed paired Student t-tests). Data are presented as mean ± SEM. (**B**) Representative superimposed AP waveforms recorded in hESC-CMs exposed to 0.1% DMSO (as vehicle control) and BF (10 nM) for 24 hours. n = 7.

To correlate the CG-induced changes in cardiac ion currents with transmembrane activities, APs were recorded in single hESC-CMs under cytosolic [Ca^2+^]_i_-independent condition (with 5 mM EGTA added in the pipette solution). At 1.0 µM, all CGs tended to shorten APDc (APD90, PAD50 and APD30) and it was more evidenced with BF and CBG. Yet only the effects of BF were statistically significant (Fig. 5A). Moreover, a moderate reductions of AP amplitude (APA) and a more depolarized shift of the maximal diastolic potential (MDP) were observed with BF and CBG, yet only BF-induced APA reduction and CBG-induced positive shift of MDP were significant (Fig. 5A). No changes in the maximal upstroke velocity (dV/dt_Max_) and beating frequency were noted. BF (10 µM) treatment for 1–2 minutes achieved more remarkable changes in APs, followed by polymorphic arrhythmia-like AP waveform at 5 minutes and AP firing ceased at 5~8 minutes (data not shown).

### Effects of CGs on cardiac field potentials of hESC-CMs

**Figure 6:**
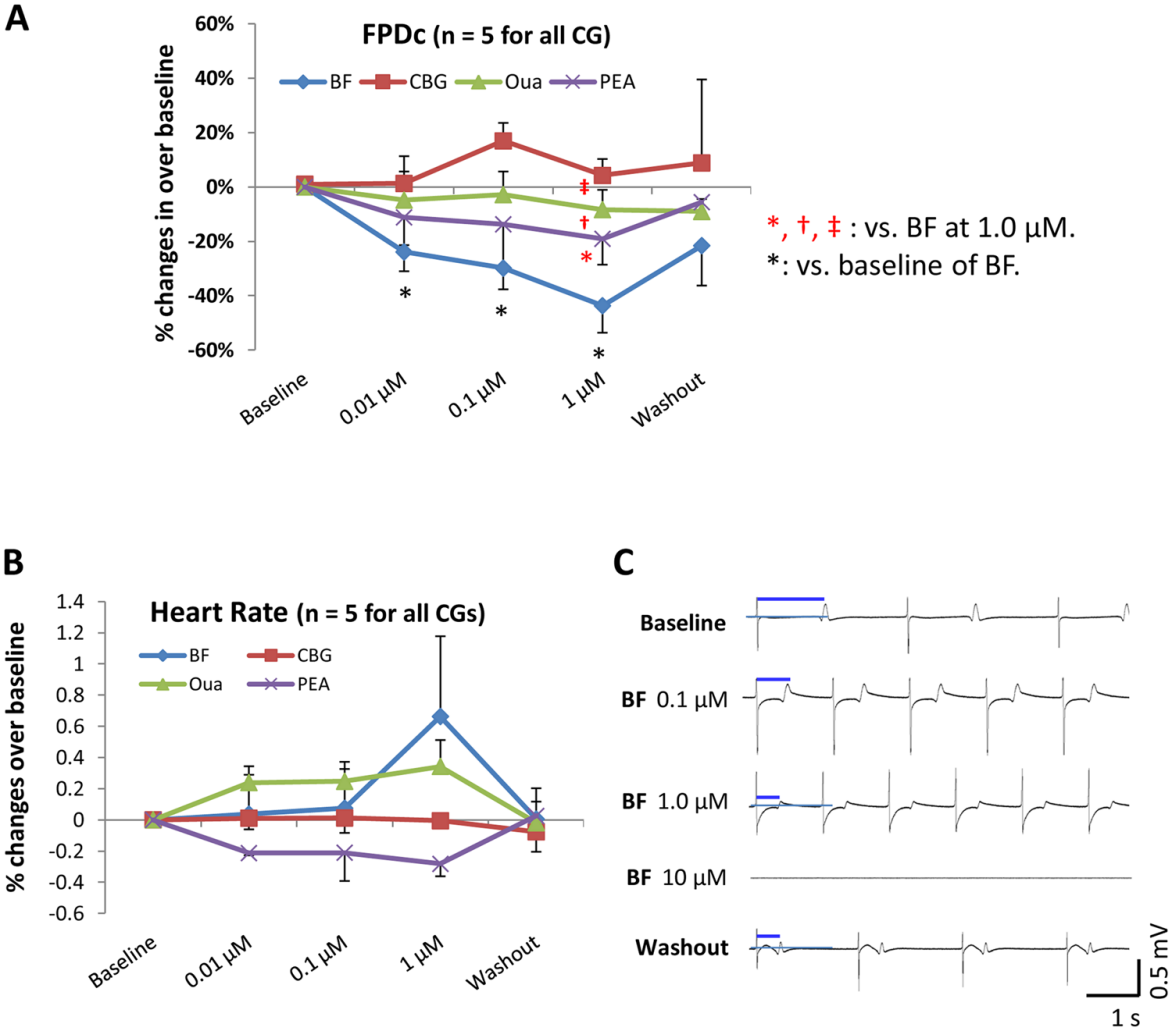
Effect of CGs on FPs in hESC-CMs. Effects of BF, CBG, Oua and PEA on cardiac FPs were recorded in clusters of hESC-CMs. (**A**) % changes of the FPDc over baseline. (**B**) % changes of the heart rate over baseline. (**C**) Representative FP traces show the effects of BF. All recordings were started after stabilization for 5 minutes. *p < 0.05, vs. baseline (One-Way ANOVA). ^*(red)^ *p* < 0.05, ^†(red)^ *p* < 0.01, ^‡(red)^ *p* < 0.001, vs. BF (Two-Way ANOVA). Data are presented as mean ± SEM.

Effects of CGs on the transmembrane activity of hESC-CMs were further tested by recording the extracellular FP of hESC-CM clusters without altering the cytosolic Ca^2+^. A trend of decreasing FP duration (FPD) by BF, Oua and PEA was noticed while only BF exerted a significant (*p* < 0.05) concentration-dependent (0.01~1 µM) effect (Fig. 6A,C). Moreover, BF and Oua tended to increase the beating frequencies yet the changes were insignificant (Fig. 6B). At 10 µM, all CGs stopped the beating of hESC-CMs in 3~5 minutes (data not shown) and the beating resumed after washout (data not shown).

### Effects of CGs on APs of zebrafish

**Figure 7:**
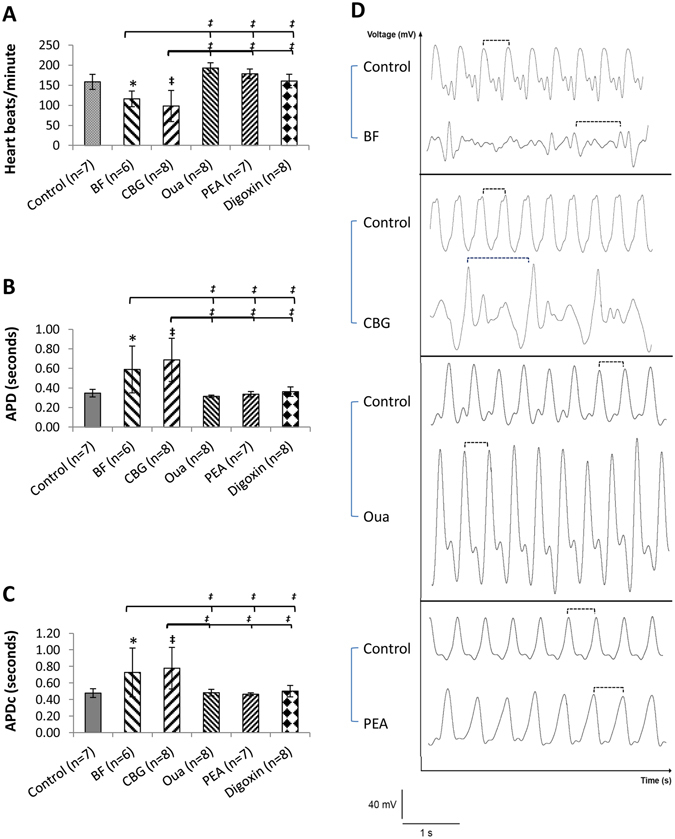
Effect of CGs on the APs of Zebrafish larva. Effects of BF, CBG, Oua and PEA (all at 100 µM) on the APs, controlled by digoxin, were analysed in day-3 Zebrafish larva. (**A**, **B** and **C**) Bar-graphs show the effects of various CGs on heart rates, APD and APDc, respectively. (**D**) Representative waveforms of APs recorded. The dashed lines indicate the representative APDs. *p < 0.05, ^‡^ *p* < 0.001, vs. control; ^‡^ *p* < 0.001, vs. BF or CBG (One-Way ANOVA). Data are presented as mean ± SD.

As all CGs at 10 µM abolished the transmembrane electrical activity *in vitro* in hESC-CMs, the effects of higher doses of CGs were tested in day-3 zebrafish larva which demonstrated insensitivities to CGs indicated by retained normal heart rhythm and APs after exposing to up to 10 µM of all CGs. However, zebrafish larvae responded to 100 µM BF and CBG demonstrated decreased heart rates (by ~27% and ~38%, respectively), prolonged APDc (by ~52% and ~63%, respectively) and early-after depolarization and polymorphic arrhythmia-like changes (Fig. 7). Effects of BF and CBG are significantly distinguishable from that of Oua, PEA and digoxin (used as a control) which showed little effect on heart rate and APDc (Fig. 7).

### Prolonged effects of CGs on action potentials of hESC-CMs

A previous study has demonstrated that CGs such as digoxin are able to delay cardiac repolarization at nanomolar concentrations associated with inhibitions of the expression and trafficking of hERG^25^. We tested the prolonged effects of 10 nM BF on APs in hESC-CMs. Cells treated with BF ≤ 10 nM for 24 hours maintained rhythmic contractions. The APs showed moderate yet significant prolongation in APD90 (Fig. 5B).

### Simulated effects of reduced Na+/K+ pump current (*I*_NaK_), *I*_Ca,L_ and *I*_Kr_ on APs of ventricular myocytes

**Figure 8:**
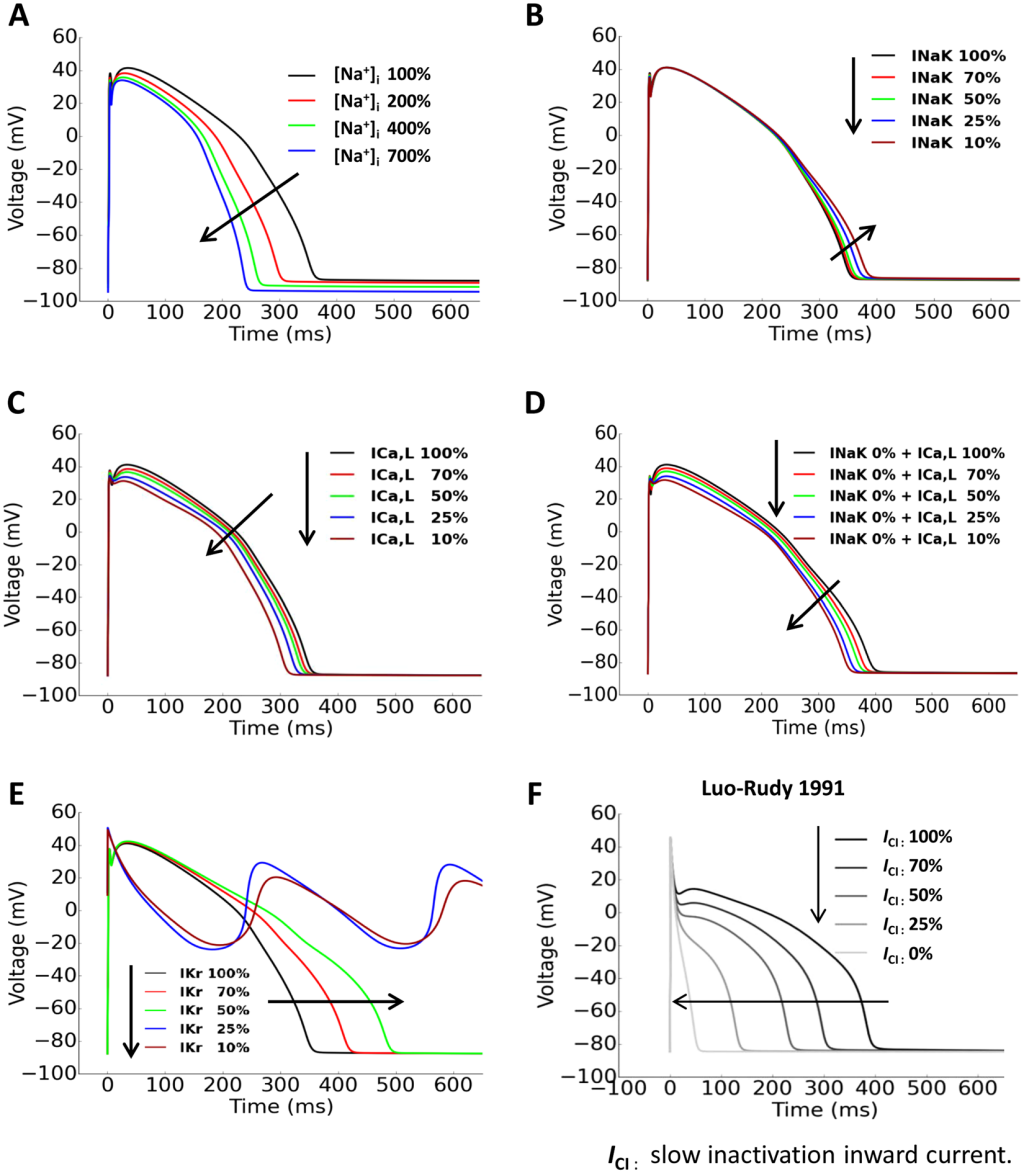
Simulated effects of reduced *I*_NaK_, *I*_Ca,L_ and *I*_Kr_ on ventricular APs. ORd model was adopted in A~E. (**A**) Simulated effect of increased [Na^+^]_i_ on APs. (**B**) Simulated effect of reduced *I*_NaK_ alone on APs. (**C**) Simulated effect of reduced *I*_Ca,L_ alone on APs. (**D**) Simulated effects of *I*_NaK_ (0%) in combination with reduced *I*_Ca,L_ on APs. (**E**) Simulated effect of reduced *I*_Kr_ on APs. (**F**) The effect of reduced *I*_Ca,L_ on APs simulated by the LRd model.

The O’Hara-Rudy (ORd) model (2011) of non-diseased human midmyocardial ventricular myocytes^26^ and the Luo-Rudy (LRd) model (1991) of guinea-pig ventricular myocytes^27^ were adopted to validate the impacts of CG-induced ion current changes on APs. The ORd model showed that increased [Na^+^]_i_ was able to reduce APD (Fig. 8A). Yet reduced *I*_NaK_ (70%, 50%, 25% and 10% of control) alone failed to positively shift MDP and shorten APD (Fig. 8B). Next, reduced *I*_Ca,L_ (70%, 50%, 25% and 10% of control) alone (Fig. 8C) or with 0% *I*_NaK_ (Fig. 8D) led to marginal/moderate decrease in APDs. On the other hand, the simulated effects of reduced *I*_Kr_ (70%, 50%, 25% and 10% of control) on APs confirmed the experimental findings (Fig. 8E). Interestingly, the LRd model revealed marked APD shortening caused by reduced (70%, 50%, 25% and 10% of control) slow inactivation inward current (*I*_CI_), an potential equivalent of *I*_Ca,L_ (Fig. 8F).

## Discussion

The current study identified the NKA inhibition-independent proarrhythmic mechanisms of CGs which involve key cardiac ion channel currents and transmembrane potentials. With the cytosolic Ca^2+^ largely chelated or extracellular Ca^2+^ depleted, bufadienolides demonstrated a cytosolic [Ca^2+^]_i_-independent *I*_Ca,L_ inhibition, accompanied by APD shortenings. With the NKA activities completely inhibited by Oua, effects of bufadienolides on *I*_Ca,L_ persisted. Further, a strong *I*_kr_ blockage in hERG-HEK cells and a prolonged APD in zebrafish larva were observed by BF and CBG at 100 µM, whereas prolonged APD was also observed in hESC-CMs treated with 10 nM BF for 24 hours. Lastly, results from computer simulation largely validated the experimental findings on the relationship between ion currents and APs.

Data from the current study indicate that NKA inhibition-independent *I*_Ca,L_ inhibition could contribute to the greater proarrhythmic effects of BF and CBG compared with Oua and PEA. While it is known that 10 μM of Oua could totally blocked the NKA activities^20, 22^ and BF and CBG are 4~7 folds more potent NKA blockers than Oua^23^, the more potent proarrhythmic effects of BF in APD shortening^13^ and [Ca^2+^]_i_ transient suppression over Oua suggesting that bufadienolides could have NKA-independent proarrhythmic effects^20, 22^. In the current study, we firstly observed cytosolic [Ca^2+^]_i_- or CDI- independent effects of BF and CBG on *I*_Ca,L_ inhibition and APD shortening. *I*_Ca,L_ was measured in the presence of 10 ~20 mM of EGTA in the pipette/internal solution and the APs were measured with 10 mM EGTA in the pipette solution, while ≥5 mM EGTA is sufficient to achieve a complete chelation of cytosolic free Ca^2+^ with [Ca^2+^]_i_ up to 2.5 mM [Calculated by a Ca-EGTA Calculator v1.3 online from Maxchelator program of Stanford]. Hence EGTA could eliminate CDI due to CG-induced NKA inhibition^28, 29^. In addition, BAPTA, the other calcium chelator we added to the intracellular solution (5 mM), could, by itself, fully block CDI^29^. Furthermore, our data showed that the *I*_Ca,L_ inhibitory effect of BF retained in Cav1.2-CHO cells with extracellular Ca^2+^ replaced by Ba^2+^, while depletion of extracellular Ca^2+^ is known to fully blocked CDI^29^. Secondly, we gathered evident to support that BF and CBG possess NKA inhibition-independent effects. The concentration-dependent *I*_Ca,L_ inhibition by BF and CBG peaked at 100 µM and it further suggests that BF and CBG are capable of inducing NKA inhibition-independent proarrhythmic effects as <2.5 µM of BF or CBG could be sufficient to fully block the NKA activities^18, 22–24^. Next, we noticed that the effects of BF and CBG on *I*_Ca,L_ and [Ca^2+^]_i_ transient persisted in cells incubated with 10 µM Oua. The rapid inhibition of *I*_Ca,L_ and shortening of APs by BF and CBG support direct effects on LTCC since the effects of NKA inhibition and CDI on APs could take longer time^13^. While CDI of *I*_Ca,L_ is known to accelerate the decay of currents without supressing the peak currents, our finding that BF, without/with NKA activity blocked by Oua, markedly inhibit peak *I*_Ca,L_ with magnitudes greater than that for the decay current (Fig. 3Bc), further support the notion that CGs are capable of NKA inhibition- and CDI- independent *I*_Ca,L_ inhibition.

*I*_kr_ blocking by CF and CBG offers an additional support to the NKA inhibition-independent proarrhythmic mechanisms of CGs. Our data show that BF and CBG at 100 µM demonstrated a strong and unprecedented *I*_kr_ blocking (>40%) which could be behind the APD prolongation in zebrafish lava. However, the impact of a potential *I*_Ca,L_ inhibition, in completion with *I*_Kr_ inhibition, on APD in Zebra fish remains unclear. Result from computer simulation may suggest that APD is more sensitive to *I*_Kr_ inhibition. It appears that zebrafish is less sensitive to CG-induced cardiotoxicity probably due to inter-species variations and the arrhythmogenic concentration of CGs is less physiologically relevant. With more clinical relevant concentrations (the recommended digoxin concentrations for the treatment of chronic heart failure are around 0.8 ng/ml (1.2 nM) with a half-life of 36 hours and the toxic level is more than 2 ng/ml^5, 30^), we demonstrated that CGs could prolongation APD and such an effect could be attributed to the chronic effects of low concentration CGs on hERG inhibition^25^.

The well-documented effect of CGs on depolarizing the MDP and shortening APD in isolated cardiac myocytes^13, 14^ appeared to be less obvious in hESC-CMs (Fig. 5). This discrepancy could be due to the shorter exposure time (1–2 minutes) designed to favours the observation of the direct effects of CGs on the ion channels; a more depolarize (~60 mV) MDP^31^ in hESC-CMs compared with the −80 mV ~ −90 mV MDP in isolated human ventricular myocytes, and the EGTA (10 mM) added in the pipette solution that could suppress CDI and minimize the APD shortening effect of CGs in hESC-CMs.

Effects of CGs on APD are supported by the simulated results in principle although only moderate APD shortening was simulated by the ORd human ventricular myocyte model (2011) in responds to a marked *I*_Ca,L_ reduction. Such a discrepancy may reflect the limitations with the ORd model, which has been shown failing to simulate considerable changes in APs with marked inhibitions of *I*_NaK_ and *I*_Ca, L_^32^. Compared with the moderate negative correlation between [Na^+^]_i_ and APDs simulated by the ORd model, Grandi *et al*.^33^, using a mathematical model for Ca^2+^ handling and ionic currents in the human ventricular myocyte, demonstrated that Na^+^ loading could be a major determinant of ventricular APD shortening, and therefore that model could be a better choice for modeling effects of NKA block and I_Ca,L_ block.

While the arrhythmogenic potential associated with LTCC inhibition could be less understood compared with hERG inhibition, the association between reduced *I*_Ca,L_ and cardiac arrhythmias is supported by the clinical findings. For example, loss-of-function mutations in the LTCC have been associated with QT shortening and severe arrhythmia in patients with the Brugada syndrome^34^.

Overall, our data may explain the dissociation between the positive inotropic effect and the proarrhythmic effects of CGs. For example, the additional LTCC inhibition by BF over Oua may undermine the hypertonic effect of BF by attenuating the [Ca^2+^]i and accelerate the proarrhythmic effects in the meantime.

## Conclusions

We collected evidence to support that bufadienolides such as BF possess NKA inhibition-independent proarrhythmic effects associated with LTCC and hERG blocking.

## Materials and Methods

### CG compounds

The structure formulas of BF, CBG, Oua and PEA are shown in Fig. 1. BF, CBG and PEA were prepared in our laboratory at Jinan University, Guangdong, China. BF and CBG were isolated and purified (>99%) from the traditional Chinese medicine Chan Su derived from toad venom^35^ while PEA was isolated and purified (>99%) from the plant *Asclepias curassavica* L. The quality of BF, CBG and PEA were confirmed by 1D NMR spectra assay. **BF**: ^1^H NMR data (CDCl_3_, 400 MHz) δ_H_ 7.83 (1H, dd, J = 9.7, 2.6 Hz), 7.22 (1H, br d, 2.6 Hz), 6.25 (1H, d, 9.7 Hz), 4.13 (1H, br s), 2.46 (1H, dd, J = 9.6, 6.5 Hz), 0.95 (3H, s), 0.70 (3H, s); ^13^C NMR data (CDCl_3_, 100 MHz) δ_C_ 162.3, 148.5, 146.7, 122.7, 115.3, 85.4, 66.8, 51.3, 48.4, 42.4, 40.9, 36.0, 35.7, 35.4, 33.3, 32.7, 29.7, 28.7, 27.9, 26.5, 23.7, 21.4, 21.4, 16.5. **CBG**: ^1^H NMR data (CDCl_3_, 400 MHz) δ_H_ 7.89 (1H, m), 7.16 (1H, br s), 6.20 (1H, dd, *J* = 9.8, 0.8 Hz), 5.46 (1H, dd, J = 9.3, 1.4 Hz), 4.14 (1H, br s)3.64 (1H, br s), 2.78 (1H, d, J = 9.3 Hz), 1.89 (3H, s), 0.99 (3H, s), 0.82 (3H, s); ^13^C NMR data (CDCl_3_, 100 MHz) δ_C_ 170.1, 161.6, 151.3, 148.3, 116.1, 113.9, 74.8, 72.8, 66.5, 59.5, 50.4, 45.2, 40.1, 39.3, 35.9, 35.5, 33.3, 33.1, 29.5, 27.9, 25.6, 22.1, 20.9, 20.6, 20.5, 17.2. **PEA**: ^1^H NMR data (pyridine-d_6_, 300 MHz) δ_H_ 10.00 (1H, s), 6.13 (1H, s), 5.28 (1H, dd, J = 18.2, 1.3 Hz), 5.03 (1H, dd, J = 18.1, 1.4 Hz), 5.02 (1H, s), 4.44 (1H, m), 4.33 (1H, m), 4.14 (1H, dd, J = 11.5, 5.2 Hz), 3.77 (1H, m), 1.38 (3H, d, J = 6.1 Hz), 0.91(3H, s); ^13^C NMR data (pyridine-d_6_, 75 MHz) δ_C_ 208.6, 176.3, 175.1, 118.2, 97.8, 93.3, 84.6, 74.4, 74.3, 72.9, 69.9, 69.1, 53.4, 51.7, 50.3, 50.2, 49.2, 43.9, 43.1, 40.4, 39.7, 36.9, 34.5, 33.0, 28.5, 27.7, 22.8, 22.1, 16.4. Oua and digoxin were purchased from Sigma-Aldrich (St. Louis, MO, USA). BF, CBG, and PEA stocks were made using DMSO whereas Oua and digoxin stocks were prepared in distilled water. The stocks were diluted using external solutions or cultural medium (for MEA assay) to make up the final concentrations for recording. The final concentration of DMSO is ≤0.2%.

### Chemicals

Unless specified, all reagents for electrophysiology assays, including Bay K8644, were obtained from Sigma-Aldrich (St. Louis, USA).

### Cells

Heterologous expressing systems were adopted for evaluating the effects of CGs on major voltage-gated cardiac ion channel currents, including the repolarising rapid component of the outward rectifier potassium current (*I*_Kr_), depolarizing peak sodium current (*I*_Na_) and depolarizing *I*_Ca,L_. Stably expressing cell lines included: hERG-HEK293 as HEK293 cells expressing human *KCNH2* or Ether-a’-go-go-Related gene (hERG) which encode hKv11.1 channel for *I*_kr_ (Chan Test, Cleveland, USA); SCN5A-HEK293 as HEK293 cells expressing human *SCN5A* and *SCN1B* which encodes hNav1.5/β1 subunit of sodium channels for *I*_Na_ (Anaxon AG, Berne, Switzerland) and Cav1.2-CHO as CHO cells expressing human *CACNA1C/CACNB2/CACNA2D1* genes which encodes hCav1.2/β2/α2δ1 channel or LTCC for *I*_Ca,L_ (ChanTest, Cleveland, USA).

hESC-CMs were adopted as the most relevant *in vitro* human model for drug testing. H3 hESCs (WiCell Research Institute, Madison, USA) were differentiated into cardiomyocytes following the published protocols^31, 36^. Cells were maintained at 37 °C in a humidified CO_2_ (5%) incubator in RPMI 1640 Medium containing 2% of B-27® supplement (+Insulin, 1 mL/50 mL) and 1% of Penicillin-Streptomycin-Glutamin (0.5 mL/50 mL), all from Invitrogen (Singapore). hESC-CMs 30~35 days post differentiation were used for MEA and AP recordings.

### Cardiac ion channel currents measurement by automated patch-clamping

Effects of CGs on *I*_Kr_, *I*_Na_ and *I*_Ca,L_ were measured in hERG-HEK293, SCN5A-HEK293 and Cav1.2-CHO cells, respectively, at room temperature by Patchliner® automated patch-clamping system (Nanion Technologies, Munich, Germany), an automated gigaseal patch clamp instrument^37^. The internal solution for measuring *I*_Na_ and *I*_Ca,L_ contained (in mM): CsCl 50, NaCl 10, Cs-Fluoride 60, EGTA 20, HEPES 10, adjusted to pH 7.20 with CsOH. To prevent rundown when recording calcium channels (in mM), Na_3_GTP 0.3, ATP (Mg salt) 5 and BAPTA (free acid) 5, were added into the *I*_Ca,L_ internal solution and adjusted to pH 7.20 with CsOH. The internal solution for measuring *I*_Kr_ contained (in mM): KCl 50, NaCl 10, K-Fluoride 60, EGTA 20, HEPES 10, adjusted to pH 7.20 with KOH. The external solution for measuring *I*_Kr_ and *I*_Ca,L_ contained (in mM): NaCl 140, KCl 4, MgCl_2_ 1, CaCl_2_ 2, glucose monohydrate 5, HEPES 10, adjusted to pH 7.40 with NaOH. The external solution for measuring *I*_Na_, contained (in mM): NaCl 80, KCl 4, MgCl_2_ 1, CaCl_2_ 2, glucose monohydrate 5, NMDG 60 and HEPES 10, adjusted to pH 7.40 with NaOH. The seal enhancer solution for increasing the probability of giga-seal formation contained (in mM): NaCl 80, KCl 3, MgCl_2_ 10, CaCl_2_ 35, HEPES (Na^+^-salt) 10, adjusted to pH 7.40 with HCl. Data was acquired using PatchMaster v2 × 65 (HEKA Elektronik, Germany) and analyzed using Igor Pro 6.37.

Two-step voltage protocols were adopted for recording *I*_Ca,L_, *I*_Kr_ and *I*_Na_ while the corresponding ion currents were evoked at the 2^nd^ step. *I*_Ca,L_ was recorded by depolarization of the cell membrane potential from a holding potential of −80 mV (50 ms) to +10 mV and held for 100 ms. *I*_kr_ was recorded by a 1^st^ pulse (+30 mV for 2000 ms) followed by a 2^nd^ pulse at voltage −50 mV for 4000 ms to evoke the *I*_Kr_ peak tail currents. *I*_Na_ was measured by clamping the cells from a holding potential of −120 mV (5 ms) to 0 mV for 10 ms. The *I*_Ca,L_, *I*_Na_ and *I*_Kr_ were validated by corresponding blockers (positive controls) which achieved full blocks.

### Conventional patch-clamp recordings

Whole cell configuration of the patch-clamp technique was used to measure *I*_Ca,L_ in Cav1.2-CHO and hESC-CMs and APs in hESC-CMs^31^. The signal was amplified using an Axopatch 700B patch clamp amplifier (Molecular Devices, Foster City, USA) and low-pass filtered at 5 kHz. Patch pipettes were fabricated from glass capillaries using a Sutter P-97 microelectrode puller (Novato, CA, USA) and the tips were heat polished with a microforge (NARISHIGE MF-900, Tokyo, Japan) to gain a resistance of 2–4 MΩ. The electrical signals were sampled at 2.5–10 kHz and filtered at 2 kHz using a low-pass filter. Data acquisition was achieved using the Digidata 1440 A (Axon Instrument). Data analysis and fit were performed using clamp fit 10.2 (Axon Instrument) and Origin 7.0 software (Origin Lab Corporation). A pClamp software (Version8.1; Axon Instrument) was used to generate voltage-pulse protocols, acquire and analyze data.

### I_Ca,L_ recording in Cav1.2-CHO

To verify the CDI-independent effects of BF, CaCl_2_ in the extracellular solution was replaced by BaCl_2_ so to let Ba^2+^ ions carried the current through Ca^2+^ channels^38^.

### I_Ca,L_ recording in hESC-CMs

I_Ca,L_ was recorded in hESC-CMs as previously described^38^. Patch pipettes solution contained (in mM): CsCl 120, MgCl_2_ 3, MgATP 5, EGTA 10, and HEPES 5, adjusted to pH 7.2 with CsOH. External solution contained (in mM): NaCl 140, CsCl 10, CaCl_2_ 1.8, MgCl_2_ 1, glucose 10, and HEPES 10, adjusted to pH 7.4 with NaOH. To eliminate the ‘run-down’ effect during *I*_Ca,L_ recordings, Ba^2+^ was also used in the external solution (BaCl_2_ 1.8 mM) to replace Ca^2+^ as charge carrier of calcium channel current^11^. Current-Voltage curve were generated by voltage clamp protocols consisting of V_hold_ = −80 mV followed by a 3 s long pre-pulse at −50 mV to inactivate Na^+^ and T-type Ca^2+^ channels, then a family of 300 ms depolarization from −50 mV to 50 mV in 10 mV increments.

Calcium channel current densities were obtained by dividing current amplitudes by membrane capacitances. Steady state inactivation variables of *I*_Ca,L_ were determined using a two-pulse gapped protocol. Potential was held at −40 mV, then pulsed to a conditioning pre-pulse ranging from −80 mV to +10 mV for 2000 ms, returned to −40 mV for 10 ms, and stepped to 0 mV for 250 ms at 10 s intervals. Voltage-dependence of activation curve and steady state inactivation curve were fitted with Boltzman equation (G = Gmax × [1 + exp(V_1/2_ − V)/κ]^−1^), where G is the conductance at various test potentials and was calculated from the peak current according to G = I/(V − Vrev), Vrev is the reversal potential obtained by extrapolating the linear part of the I/V curve to its intersection with the voltage axis. Gmax is maximum conductance; V_1/2_ and κ are half-activation voltage and the slope factor. The concentration-response data were fitted with Hill equation: I/Imax = 1/[1+ (D/IC_50_)^n^], where I is the peak current in various concentrations of compound, Imax is the maximal peak current, D is the compound concentration, IC_50_ is the drug concentration for 50% inhibition, and n is the Hill coefficient.

The time course of recovery from inactivation of *I*_Ca,L_ was studied using a two-pulse protocol: a 250-ms pre-pulse (P1) at 0 mV from the holding potential of −50 mV followed by a variable recovery period and a 250-ms test pulse (P2) at 0 mV to assess the amount of current recovered. Each two-pulse sequence was separated by a 30 s interval. The time course of recovery for *I*_Ca,L_ was determined by fitting the data points to a single exponential function: I/Imax = 1 − exp(−t/τ), where Imax and I were the peak current at pre-pulse (P1) and test pulse (P2), respectively; t was the variable recovery time; τ was the recovery time constant.

### AP measurement in hESC-CMs

APs were recorded under current-clamp mode in normal Tyrode’s solution contained (in mM): NaCl 140, KCl 5.4, CaCl_2_ 1.8, MgCl_2_ 1, glucose 10, HEPES 10, adjusted to pH 7.4 with NaOH. Pipette solution contained (in mM): KCl 130, NaCl 5, MgCl_2_ 1, MgATP 3, EGTA 10, and HEPES 10, adjusted to pH 7.2 with KOH. The parameters of APs include APD at 30%, 50% and 90% of repolarization (APD30, APD50, and APD90), APA, MDP, and beating frequency were analyzed^31, 38^. Cells were maintained at 35 °C by a temperature controller (Warner Instruments, Hamden, USA) during the recording of APs. The APDs were corrected by the beating frequency (APDc) with Fridericia’s formula (APDc = APD/interspike interval^1/3^)^39^.

### Laser-scanning confocal calcium imaging

[Ca^2+^]_i_ transients were recorded in hESC-CMs using a LSM-710 laser scanning confocal microscope (Carl Zeiss, Inc, Germany) with a 40×, 1.3 numerical aperture oil immersion objective and axial resolutions of 1.5 µm^38^. Briefly, hESC-CMs were loaded with 2 μM Fluo-8 AM (AAT Bioquest, Inc. Sunnyvale, CA, USA) for 15 min at 37 °C, and recorded in normal Tyrode’s solution. Fluo-8 was excited at 488 nm, and fluorescence emission was measured at 505 nm. Images were acquired in the line-scan (X-T) mode with 512 pixels (pixel intervals of 0.15 μm) per line at a rate of 3 ms per scan. The [Ca^2+^]_i_ transients were analyzed using a modified version of MATLAB program. The Ca^2+^ fluorescence emission intensity was expressed as F/F_0_ where the F_0_ was the basal fluorescence intensity level. The recording was performed at 35 °C.

### Multi-electrode Arrays

The extracellular FP produced by hESC-CMs was measured by multi-electrode array (MEA) assay using Multi Channel Systems MCS GmbH (Aspenhaustrasse, Reutlingen, Germany). Clusters of contracting hESC-CMs were plated on Matrigel® coated MEA chips containing 59 titanium electrodes and 1 internal reference electrode. Stocks of CGs were diluted to various concentrations in cell cultural medium (RPMI1640 basal medium). Data was acquired with an interval of 5 minutes at baseline and post drug applications. The FPD was analysed and corrected by the beating frequency (FPDc) with Fridericia’s formula (FPDc = FPD/interspike interval^1/3^)^39^.

### Cardiac AP measurement in zebrafish

Wild-type zebrafish (AB, ZIRC) were maintained as described^40^. All animal experiments were carried according to the regulations of Institutional Animal Care and Use Committee (Biological Resource Center of Biopolis of Singapore, license no. 120787), which approved this study. Developmental stages are in hours post fertilization (hpf) at 28.5 °C^41^.

Micropipettes for AP measurement on whole zebrafish larvae were prepared by pulling fire-polished borosilicate glass capillaries (World Precision Instruments) using the Flaming/brown micropipette puller P-1000 (Sutter Instrument). The micropipette was filled with internal buffer contained (in mM): NaCl 174, KCl 2.1, MgSO_4_.7H_2_0 1.2, Ca(NO_3_)_2_.4H_2_O 1.8, HEPES 15, adjusted to pH 7.2. The micropipette tip was positioned right above the pericardial region of the zebrafish heart. The electrical signals were recorded by pCLAMP 10 software (Molecular Devices) after amplification via Multiclamp 700B amplifier (Molecular Devices, Foster City, USA) and digitization through Axon Digidata 1440A digitizer (Molecular Devices). Data were analysed with Clampfit 10 software (Molecular Devices). For controls, the zebrafish larvae were mounted (laterally) in 1% low melting agarose in a glass dish and submerged in external buffer: 1X Egg water (0.6 g/L sea salt in reverse osmosis purified water). To assess the effect of drugs on the heart activity of zebrafish, the mounted larvae are pre-treated with the various drugs (diluted in1X Egg water). APDs were determined by measuring the adjacent peaks of the action potentials using the pClamp software. APDs were corrected by heart rate as above-mentioned.

### Computer simulation

O’Hara-Rudy (ORd) model (2011) of non-diseased human midmyocardial ventricular myocytes^26^ and Luo-Rody (LRd) model (1991) of mammalian (Guinea-pig) ventricular myocytes^27^ were adopted. The cycle length was 1 s.

### Statistical analysis

Numerical data are presented as mean ± standard division (SD) or mean ± standard error of mean (SEM). Comparisons were made with paired and unpaired (two-tailed) Student t-test, One-Way repeated measures ANOVA followed by the Tukey’s post hoc testing and Two-Way repeated measures ANOVA followed by the Bonferroni post hoc testing using GraphPad Prism 5.0 (GraphPad Prism 5.0, La Jolla, USA). A *p*-value of <0.05 was considered statistically significant.
