# Erratum: Identification of Na^+^/K^+^-ATPase inhibition-independent proarrhythmic ionic mechanisms of cardiac glycosides

**DOI:** 10.1038/s41598-017-09613-3

**Published:** 2017-09-13

**Authors:** Cai Hong Koh, Jianjun Wu, Ying Ying Chung, Zhenfeng Liu, Rong-Rong Zhang, Ketpin Chong, Vladimir Korzh, Sherwin Ting, Steve Oh, Winston Shim, Hai-Yan Tian, Heming Wei

**Affiliations:** 1 National Heart Research Institute Singapore, National Heart Centre Singapore, Singapore, 169609 Republic of Singapore; 2 Guangdong Province Key Laboratory of Pharmacodynamic Constituents of TCM and New Drugs Research, College of Pharmacy, Jinan University, Guangzhou, 510632 P.R. China; 3 Zebrafish Translational Unit, Institute of Molecular and Cell Biology, Singapore, 138673 Republic of Singapore; 4 International Institute of Molecular and Cell Biology in Warsaw, 4 Ks. Trojdena Street, 02-109 Warsaw, Poland; 5 Bioprocessing Technology Institute, A*STAR (Agency for Science, Technology and Research), Singapore, 138668 Singapore; 6 Cardiovascular & Metabolic Disorders Program, Duke-NUS Medical School Singapore, Singapore, 169857 Republic of Singapore

*Scientific Reports* **7**:2465; doi:10.1038/s41598-017-02496-4; Article published online 26 May 2017

The original version of this Article contained errors in the title. “Electronic supplementary materialIdentification of a Na^+^/K^+^-ATPase inhibition-independent proarrhythmic ionic mechanisms of cardiac glycosides”.

now reads:

“Identification of Na^+^/K^+^-ATPase inhibition-independent proarrhythmic ionic mechanisms of cardiac glycosides”. These errors have now been corrected in the PDF and HTML versions of the Article.

